# Supplementary material for: Splice-Junction-Based Mapping of Alternative Isoforms in the Human Proteome
Source: Cell Rep. Author manuscript; Available in PMC 2020 Jan 15. (PMC6961840; doi:10.1016/j.celrep.2019.11.026)
Supplement: 2 [file NIHMS1546469-supplement-2.zip › DF1/PXD010154/Lung/Lung_11_FLNA_FGGEHVPNSPFQVTAPERPLVGVNGLDVTSLRPFDLVIPFTIK.pdf]

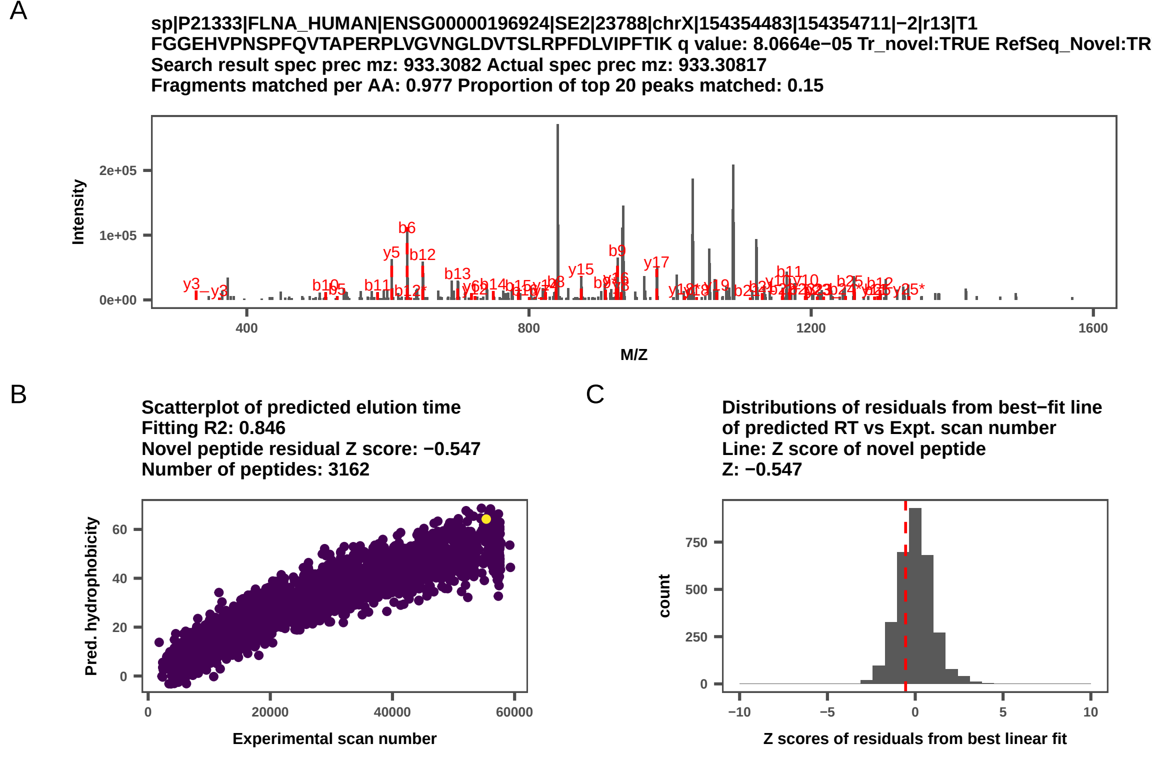
A
sp|P21333|FLNA_HUMAN|ENSG00000196924|SE2|23788|chrX|154354483|154354711|−2|r13|T1
FGGEHVPNSPFQVTAPERPLVGVNGLDVTSLRPFDLVIPFTIK q value: 8.0664e−05 Tr_novel:TRUE RefSeq_Novel:TR
Search result spec prec mz: 933.3082 Actual spec prec mz: 933.30817
Fragments matched per AA: 0.977 Proportion of top 20 peaks matched: 0.15
Intensity
2e+05
1e+05
0e+00
b6
y5
b12
b13
y15
b9
y17
b11
y3
M/Z
400
800
1200
1600
B
Scatterplot of predicted elution time
Fitting R2: 0.846
Novel peptide residual Z score: −0.547
Number of peptides: 3162
Pred. hydrophobicity
60
40
20
0
20000
40000
60000
Experimental scan number
C
Distributions of residuals from best−fit line
of predicted RT vs Expt. scan number
Line: Z score of novel peptide
Z: −0.547
count
750
500
250
0
−10
−5
0
5
10
Z scores of residuals from best linear fit
